# Similar Gut Bacterial Microbiota in Two Fruit-Feeding Moth Pests Collected from Different Host Species and Locations

**DOI:** 10.3390/insects11120840

**Published:** 2020-11-28

**Authors:** Qiang Gong, Li-Jun Cao, Li-Na Sun, Jin-Cui Chen, Ya-Jun Gong, De-Qiang Pu, Qiong Huang, Ary Anthony Hoffmann, Shu-Jun Wei

**Affiliations:** 1 Institute of Plant and Environmental Protection, Beijing Academy of Agricultural and Forestry Sciences, 9 Shuguanghuayuan Middle Road, Haidian District, Beijing 100097, China; gqiang1995@126.com (Q.G.); gmatjhpl@163.com (L.-J.C.); sunlina19952005@163.com (L.-N.S.); chenjincui1314@126.com (J.-C.C.); gongyajun200303@163.com (Y.-J.G.); 2 Institute of Plant Protection, Sichuan Academy of Agricultural Sciences, Chengdu 610066, China; pdqpudeqiang@163.com; 3 College of Forestry, Sichuan Agricultural University, Wenjiang 611130, China; huangqiong711011@163.com; 4 Department of Forestry Protection, Beijing Forestry University, Beijing 100083, China; 5 Pest and Environmental Adaptation Research Group, School of BioSciences, Bio21 Institute, University of Melbourne, Parkville, Victoria 3052, Australia; ary@unimelb.edu.au

**Keywords:** *Carposina sasakii*, *Grapholita molesta*, gut microbiota, host, orchard

## Abstract

**Simple Summary:**

The peach fruit moth, *Carposina sasakii*, and the oriental fruit moth, *Grapholita molesta* are two co-occurring pests in orchards. Larvae of both species bore into fruits and cause damage to fruit production. Understanding the gut microbes, as well as the influencing factors between these co-occurring pests, may provide insight into their occurrence and control. In this study, we found that the two pests shared many bacteria in their gut from the genera *Pseudomonas*, *Gluconobacter*, *Acetobacter*, and *Pantoea*. The composition of the gut microbiota is similar between the two species collected from the same host plant and orchard; however, the gut microbiota of individuals collected from different orchards of the same host plant can be different within pest species. These results show that the two fruit moth pests have similar gut bacteria and varied environment in orchards can influence their gut microbiota.

**Abstract:**

Numerous gut microbes are associated with insects, but their composition remains largely unknown for many insect groups, along with factors influencing their composition. Here, we compared gut bacterial microbiota of two co-occurring agricultural pests, the peach fruit moth (PFM), *Carposina sasakii*, and the oriental fruit moth (OFM), *Grapholita molesta*, collected from different orchards and host plant species. Gut microbiota of both species was mainly composed of bacteria from Proteobacteria, followed by Firmicutes. The two species shared bacteria from the genera *Pseudomonas*, *Gluconobacter*, *Acetobacter*, and *Pantoea*. When we compared two pairs of PFM and OFM populations collected from the same host species and the same orchard, there is no difference in alpha and beta diversity in gut microbiota. When we compared gut microbiota of the same species and host plant from different orchards, alpha and beta diversity was different in populations of PFM collected from two pear orchards but not in other comparisons. Our study suggests that the two pests share many features of gut microbiota and environment in orchards is a main factor influencing their gut microbiota.

## 1. Introduction

Many microorganisms have become adapted to their insect hosts, forming close mutualistic relationships [1,2]. These microbes play important roles for their hosts, such as digestion and nutrient absorption of host food, protection against pathogens, and enhancement of immunity [3,4,5]. The study of insect microorganisms can point to new approaches for the control of agricultural pests and human disease vectors and increase the value of economically important insects, particularly by modifying the symbiotic relationship between symbionts and their hosts [6,7].

The community of microorganisms living in insects can be affected by many environmental factors [1,8,9,10]. In particular, gut bacteria of different insects can vary greatly in number, composition, distribution, and function for species adapted to different hosts and living in different habitats [11]. Moreover, there can be a dynamic interaction between bacteria living in the gut and the environment, as indicated by the acquisition and loss of *Serratia symbiotica* strains in aphids [12].

Moths include some of the most damaging agricultural and forest pests from the order Lepidoptera. It was reported that microbes in the gut of moths are extremely low-abundance and predominantly leaf derived, suggesting that microbes can be highly influenced by environments [13]. Being holometabolous, moths are characterized by different life stages and can vary in their gut microbiota during development [14,15,16]. Many moths are polyphagous, having a wide range of diets, representing some of the factors impacting bacterial communities in this group [15,17]. Moths are useful model species to understand the determinants of gut microbiota. Current studies have investigated factors that potentially influence gut microbiota in some moths, such as life stages [18], host plant, physiology, gut region, and habitat [19,20]. However, comparisons of microbiota among populations and species remain to be explored in moths.

Here, we focus on the peach fruit moth (PFM), *Carposina sasakii*, and the oriental fruit moth (OFM), *Grapholita molesta*, common agricultural moth pests damaging many economically important fruit crops, such as apple, pear, and peach [21,22,23,24]. Larvae of both these species bore into and feed on fruit, while OFM can also bore into tree shoots prior to pupation. These species usually co-occur in the same orchard and sometimes on the same fruit [25,26,27]. The concealed lifestyle and wide range of host plants used by these species make them useful to understand factors affecting their gut microbiota. Previous studies found that larvae of these two moths harbor a high diversity and richness of bacteria [28,29], but it is not yet clear the two species are more likely to share the same gut microbores when they live in the same orchard and on the same host plant species.

We examined gut bacterial microbiota in co-occurring PFM and OFM collected from the same host plant species, with the microbiota characterized using the 16S rRNA gene. We aimed to examine the relative contributions of the host plant, and other factors related to variation among orchards to microbial composition in these two moths.

## 2. Materials and Methods

### 2.1. Sample Collection and DNA Extraction

We sampled three pairs of PFM and OFM populations from the same host plant and orchard, as well as one PFM population from another apple orchard, and two OFM populations from two peach orchards infesting tree shoots (Table 1). The six orchards are from Beijing area with a distance from 18 to 110 km. No pest control measurement was conducted in orchards for collecting samples of PKPR, OKPR and PDAE, while orchards for collecting other specimens were well managed. In each orchard, we collected potentially infested pears and apples and peach shoots from the field and kept them in the conditioned laboratory under 25 ± 1 °C, 60 ± 5% humidity, and a photoperiod of 16 h light: 8 h dark. We collected fifth instar larvae when they came out from the damaged fruits or peach shoots. The specimens were identified by morphology and kept in a 1.5 mL sterile tube individually for 24 h to clean out the feces by starvation. Then, the collected larvae were frozen in liquid nitrogen and stored in a −80 °C refrigerator prior to usage. Based on our dissection of the damaged fruits, larvae of the same and different species could be from the same or different individual fruits; there was only one OFM larva in each peach shoot. We examined the gut microbiota of 19 PFM and 25 OFM individuals (Table 1).

Prior to DNA extraction, larvae were washed three times with 75% alcohol, then three times with sterile water. The whole gut tissue was dissected and homogenized in a 1.5 mL tube by grinding manually. Total DNA was extracted from single samples using the E.Z.N.A.^®^ Bacterial DNA Kit (Omega Bio-tek, Norcross, GA, U.S.) according to the manufacturer’s protocol. The concentration and quality of the extracted DNA were determined by a NanoDrop 2000 UV-vis spectrophotometer (Thermo Scientific, Wilmington, NC, USA) and gel electrophoresis on 1% agarose.

### 2.2. 16S rRNA Gene Amplification and Sequencing

We used the V3-V4 hypervariable regions of the bacterial 16S ribosomal RNA (rRNA) gene to examine the gut microbiota of PFM and OFM. A 468-bp target gene segment was amplified by primer pair of 338F (5′-ACTCCTACGGGAGGCAGCAG-3′) and 806R (5′-GGACTACHVGGGTWTCTAAT-3′) [30]. For PCR reaction, 20 μL of the mixture was prepared, including 5 x FastPfu reaction buffer, 250 μM dNTPs 1 U FastPfu Polymerase (Transgene, Beijing, China), 200 nM of each prime (Majorbio, Shanghai, China), 1 µL of template DNA and DNA-free water. The PCR reaction involved a single denaturation step at 95 °C for 3 min, followed by 27 cycles of 95 °C for 30 s, 55 °C for 30 s, 72 °C for 45 s, and finished after a final extension at 72 °C for 10 min. The PCR products were run on a 2% (*w*/*v*) agarose gel and those with correct size were excised and purified with an AxyPrep DNA gel extraction kit (Axygen Biosciences, Union City, CA, USA). Illumina Miseq sequencing libraries were constructed using the TruSeqTM DNA Sample Prep Kit (San Diego, CA, USA) for the purified 16S PCR products, and sequenced on an Illumina MiSeq (San Diego, CA, USA) to obtain 300 bp paired-end reads.

### 2.3. Quality Control and OTU Identification

Raw data from Illumina MiSeq sequencing were demultiplexed to obtain sequencing data for each sample. The quality of raw data was checked by FASTQC version 0.19.6 [31]; low-quality data were trimmed and filtered by Trimmomatic version 0.36 [32]. Paired-end reads were merged by FLASH version 1.2.11 [33] to generate unpaired longer reads with the following criteria: (i) the reads were truncated at any site receiving an average quality score < 20 over a 50 bp sliding window; (ii) primers were matched allowing two nucleotide mismatching, and reads containing ambiguous bases were removed [34]; only paired-end reads whose overlap longer than 10 bp were merged.

Operational taxonomic units (OTUs) were clustered with a 97% similarity threshold using UPARSE version 7.0.1090 [35], and chimeric sequences were identified and removed using UCHIME algorithm in USEARCH version 7.0 [36]. The taxonomy of each 16S rRNA gene sequence was analyzed by a naïve Bayesian classifier of Ribosomal Database Project version 2.11 [37] against the SILVA rRNA database [38]. To avoid the influence of varied sequencing depths among samples, sequences from different samples were rarefied based on the lowest depth among samples (value = 33359). Sample sequence extraction and species screening of OTU were conducted in accordance with the following conditions: (i) removal of mitochondrial, chloroplast, and *Wolbachia* sequences; (ii) retention of only OTUs with sequence depth greater than or equal to five in at least three samples in subsequent analyses, or OTUs with sequence depth greater than or equal to 20 in at least one sample.

### 2.4. Diversity Analysis

For alpha diversity, community richness indexes (sobs, chao, and ace) and community diversity indexes (Shannon, Simpson, and Pd) were estimated. The software Mothur [39] was used to calculate the alpha diversity index under different random sampling, and the *ggplot2* R package was used to draw the rarefaction curves. The Wilcoxon rank-sum test was used to compare statistical differences between different groups, while the Kruskal-Wallis rank-sum test was used for overall comparison to examine the species, host, and orchard effects. In the beta diversity analysis, principal coordinates analysis (PCoA) was conducted based on a Bray-Curtis dissimilarity matrix computed from the samples. For group comparisons, a non-parametric multivariate statistical test, permutational multivariate analysis of variance (PERMANOVA), was conducted based on the Bray-Curtis dissimilarity matrix, using QIIME1.9.1 and the R package *vegan* [40].

## 3. Results

### 3.1. Community Composition of the Gut Microbiota in PFM and OFM

When we kept reads of *Wolbachia*, OTUs of PFM were mainly annotated to *Wolbachia* (62.06%) at the genus level (Figure S1). Since *Wolbachia* is an endosymbiont, we removed reads of *Wolbachia* from subsequent analysis. Rarefaction curves from both the original sequencing data sets and randomly subsampled data sets showed that the curves of all samples tended to be flat at about 4000–5000 reads, indicating that the amount of sequencing data is enough to reflect most of the microbial diversity information in the samples (Figure S2). Total *Wolbachia* reads are 549,604. After removing the chloroplast, mitochondrial and *Wolbachia* sequences, the read count ranged from 712 to 66,707 per sample; the effective sequence count per sample was 385 after filtering (Table S1). In total, 239 OTU were clustered, attributed to 12 phyla and 158 genera and 208 species for both hosts, among which 87 OTUs belonging to 78 species and 199 OTUs belonging to 174 species were identified for PFM and OFM respectively (Table S2).

**Figure 1:**
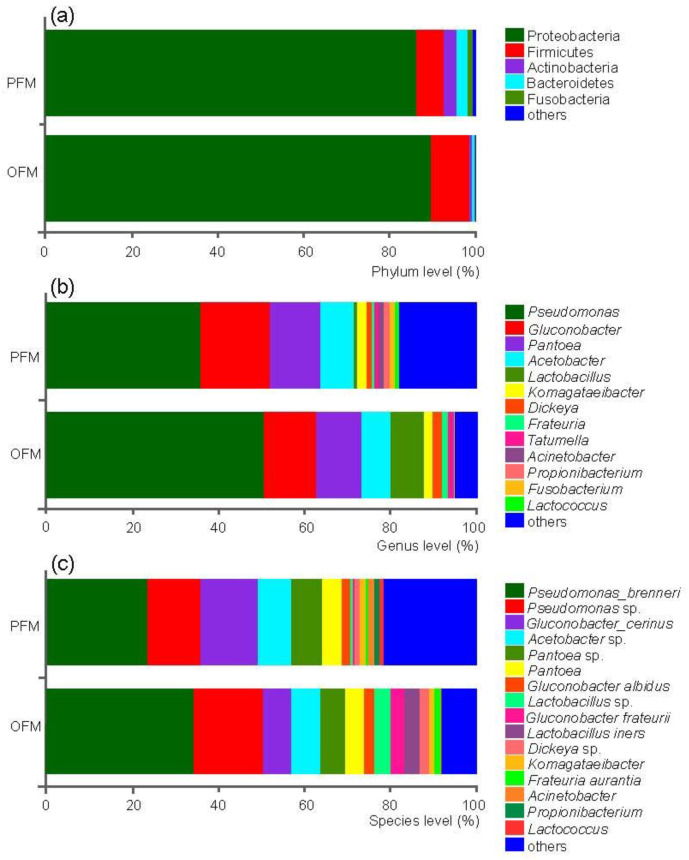
Microbial composition identified in the peach fruit moth (PFM) *Carposina sasakii* and the oriental fruit moth (OFM) *Grapholita molesta*. Community composition of the microbiome on phylum (**a**), genus (**b**), and species (**c**) levels for the PFM and OFM were illustrated. The width of the bar represents the relative abundance of each taxa.

At the phylum level, OTUs in both species were mainly attributed to Proteobacteria (86.10% in PFM, 89.54% in OFM), followed by Firmicutes (6.43% in PFM, 9.04% in OFM) (Figure 1a). At the genus level, OTUs of PFM were mainly annotated to *Proteobacteria* (35.86%), *Gluconobacter* (16.04%), *Pantoea* (11.88%) and *Acetobacter* (7.71%), while OTUs of OFM were mainly annotated to *Pseudomonas* (50.45%), *Gluconobacter* (12.15%), *Pantoea* (10.63%), *Lactobacillus* (7.84%), *Acetobacter* (6.71%) (Figure 1b). Similar patterns were found at the species level where these could be identified (Figure 1c).

**Figure 2:**
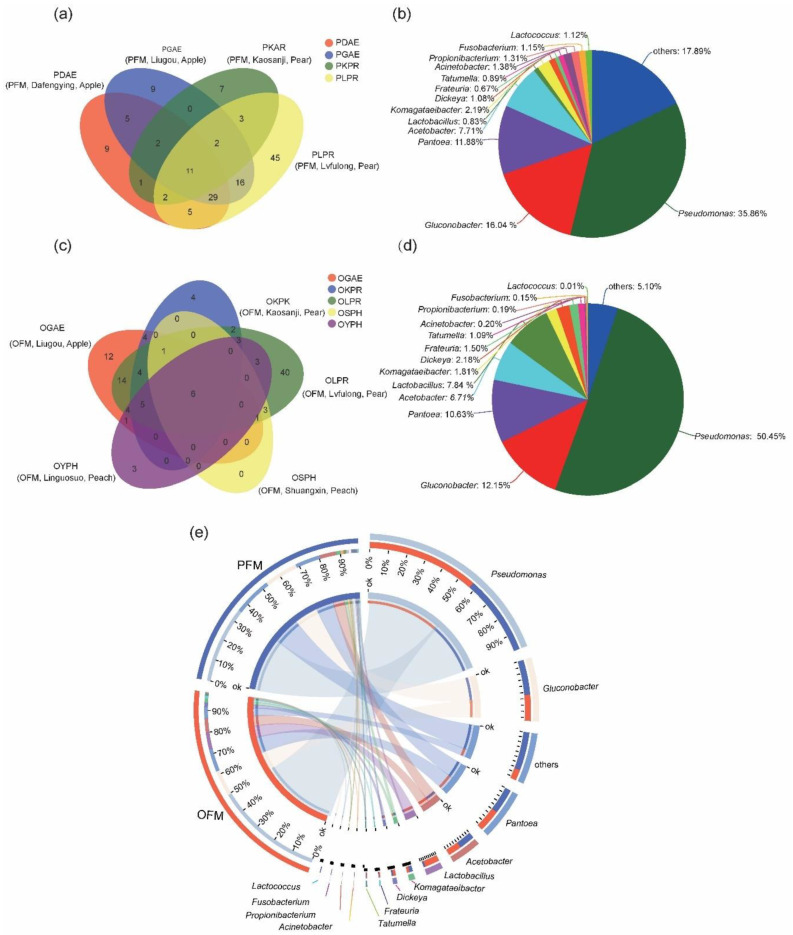
Common bacteria among populations of the same moth and between peach fruit moth (PFM) *Carposina sasakii* and the oriental fruit moth (OFM) *Grapholita molesta*. (**a**) Composition of 11 core genera found in four populations of PFM collected from different orchards. (**b**) Venn diagram at the genus level of PFM in four populations of PFM. (**c**) Composition of six core genera found in five populations of OFM collected from different orchards. (**d**) Venn diagram at the genus level of OFM from five populations of OFM. Moth species, collection location and host were provided in brackets following the four-letter sample code (Table 1). (**e**) The co-occurrence relationships of core bacteria between the PFM and OFM.

The core bacterial community at the genus level for each species was identified by comparing individuals from different orchards. For PFM, 11 core genera were identified from the four orchards sampled (Figure 2a), the most common of which were *Pseudomonas* (35.86%), followed by *Gluconobacter* (16.04%), *Acetobacter* (9.77%) and *Pantoea* (8.81%) (Figure 2b); for OFM, six core genera were identified from five orchards (Figure 2c), the most common of which were *Pseudomonas* (50.24%), followed by *Pantoea* (12.39%), *Gluconobacter* (11.29%) and *Lactobacillus* (6.87%) (Figure 2d).

In summary, Proteobacteria was the most abundant phylum for both host species. There were many common bacteria, such as the genus *Pseudomonas*, *Gluconobacter*, *Acetobacter* and *Pantoea*. In both moths, the *Pseudomonas* was the most abundant genus, followed by *Gluconobacter* and *Pantoea* (Figure 2b,d,e).

### 3.2. Comparison on Gut Microbiota between PFM and OFM

**Figure 3:**
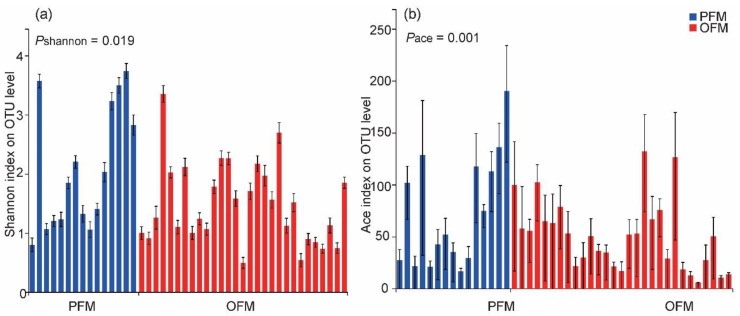
Community diversity (**a**) and richness (**b**) for the OTU level of gut bacterial microbiota between the peach fruit moth (PFM) *Carposina sasakii* and the oriental fruit moth (OFM) *Grapholita molesta*. Each bar represents an individual moth. There is significance of the difference between PFM and OFM in diversity and richness in term of Shannon (**a**) and Ace (**b**) index estimated by Kruskal–Wallis test.

When gut bacterial microbiota was compared between all samples of PFM and OFM, in terms of alpha diversity, there was significant difference in OTU diversity (*P*_shannon_ = 0.019) and richness between PFM and OFM (*P*_ace_ = 0.001) (Figure 3a,b; Table S3).

**Figure 4:**
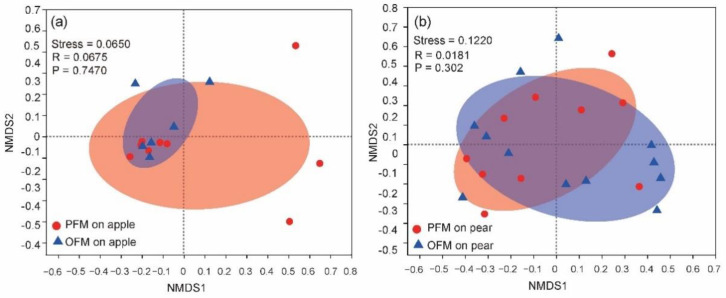
Comparison of gut bacterial microbiota between the peach fruit moth (PFM) *Carposina sasakii* and the oriental fruit moth (OFM) *Grapholita molesta* on apple (**a**) and pear (**b**). Value of the stress is less than 0.2 in both comparisons, indicates that the data can be represented by the two-dimensional dot diagram of NMDS. R is < 1, indicating that there is no significant difference between and within groups. *p* value is > 0.05, indicates there is no significant difference between PFM and OFM on either on apple or on pear.

We then compared gut bacterial microbiota between two pairs of PFM and OFM populations collected from the same host species and the same orchard. In terms of alpha diversity, there is no significant difference in diversity (*P*_shannon_ = 0.5939, *P*_simpson_ = 0.68,) and richness (*P*_ace_ = 0.5959, *P*_chao_ = 0.7234) between PFM and OFM. In terms of beta diversity, individuals of PFM and OFM from the same host did not show significant difference in the NMDS analysis (Figure 4). ANOSIM analysis showed that there is no significant difference between PFM and OFM on species level of gut bacteria when individuals of moths were grouped either considering host plant (*P* = 0.09) or not (*P* = 0.747 for apple, *P* = 0.302 for pear).

### 3.3. Influence of Orchard on Gut Microbiota within Species

**Figure 5:**
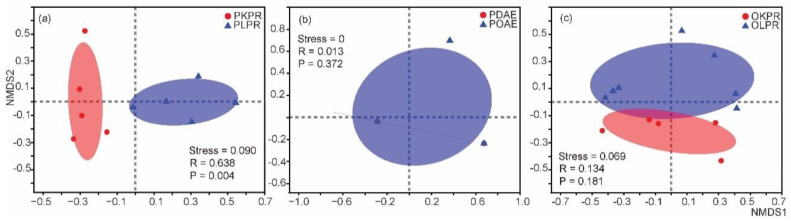
Comparison of gut bacterial microbiota for populations of peach fruit moth (PFM) *Carposina sasakii* and the oriental fruit moth (OFM) *Grapholita molesta* on pear (**a**), apple (**b**) and peach shoot (**c**). Due to the limited number of samples on peach shoot, the above beta diversity cannot be conducted for populations of OFM collected from two orchards of peach shoot.

We compared the gut microbiota of the same species and host plant from different orchards to test the effect of the orchard by fixing the host plant (Table S3). Two pairs of PFMs from pear and apple and two pairs of OFM from pear and peach shoot hosts were used for analysis. In terms of alpha diversity (Ace), significant difference in both diversity (*P*_shannon_ = 0.0216) richness (*P*_ace_ = 0.0122) was found in two PFM comparison on pear (PKPR and PLPR), while no significant difference was found in either diversity (*P*_shannon_ = 1) or richness (*P*_ace_ = 0.5403) in two PFM comparison on apple (PDAE and PGAE); there is no significant difference in either diversity or richness in comparisons of OFM on either pear (OKPR and OLPR) or peach shoot (OSPH and OYSH) except for richness of OFM on pear (*P*_ace_ = 0.0230 for OKPR and OLPR). In terms of beta diversity, significant difference between two populations of PFM collected from two orchards of pear (Figure 5a), but not between populations of PFM collected from two orchards of apple (Figure 5c), or between populations of OFM collected from two orchards of peach shoot (Figure 5c). These results suggest that orchard can affect the composition of gut microbiota in PFM and OFM.

## 4. Discussion

### 4.1. Comparison of Gut Microbiota from Two Fruit Borers

In this study, we found that the gut microbiota of PFM and OFM was dominated by Proteobacteria and Firmicutes, which is similar to the situation found in Liu et al. and Li et al. [28,29], and in other lepidopterans such as *Lymantria dispar*, *Helicoverpa armigera*, and *Bombyx mori* [41,42,43,44]. However, there was a difference between PFM and OFM and other lepidopterans at the genus level. OTUs from both PFM and OFM was dominated by *Pseudomonas*, *Gluconobacter*, *Acetobacter*, and *Pantoea*. In contrast, in silkworms, *Aureimonas*, *Methylobacterium*, *Rhizobium*, *Sphingomonas*, *Propionibacterium*, *Pseudomonas*, and *Microbacterium* were the most common genera [44]. The results suggest that PFM and OFM gut microbes had a similar composition, but they are different from those of the *Bombyx mori*, which has a different diet.

When we focused on the gut microbes of PFM and OFM from the same host and the same orchard, this pattern was also found: *Lactobacillus* was rare in PFM (0.83%) and abundant in OFM (7.84%). Perhaps this difference in species might generate phenotypic differences among the species for traits such as pesticide resistance. For instance, insecticide-treated resistant strains of the diamondback moth *Plutella xylostella* had more *Lactobacillales* and the less common taxa *Pseudomonadales* and *Xanthomonadales* as well as fewer *Enterobacteriales* compared with a susceptible strain [45]. The OFM microbiota might contribute to resistance, although living in fruit they would be less affected by pesticides than *Plutella xylostella* larvae feeding on leaves. The comparison of microbes of PFM and OFM in three orchards showed that there was no large difference in microbial richness and diversity between PFM and OFM.

### 4.2. Influence of Orchard and Host Species on Gut Microbiota

Microbial communities can vary among host locations, both in terms of community diversity and community structure [46]. In our study, there were differences in gut bacterial microbiota in larvae of the same moth collected from different orchards of pear. However, we found no difference in gut microbiota between two moths collected from the same host plant. Differences in gut microbiota have also been noted in studies on other insects, such as in comparisons of *Drosophila* between indoor and wild environments [47]. Our results are consistent with other studies showing that environmental factors are important in influencing the gut microbiomes of some caterpillars [13].

### 4.3. Wolbachia in PFM and OFM

*Wolbachia* is typically an intracellular endosymbiont rather than a gut bacterium, but it can be found in the gut wall of some species [48]. It is common in Lepidoptera [49], where its incidence across Lepidoptera is poorly understood, although in Lepidoptera it can cause a variety of effects on host reproduction including cytoplasmic incompatibility, feminization and male killing [50,51,52] and increases the susceptibility of its host to baculovirus [53]. These effects have not yet been investigated in PFM and OFM and require a comparison of *Wolbachia* infected and uninfected individuals for fitness as well as crosses to establish reproductive effects. Of particular interest from the perspective of the current study is whether *Wolbachia* might influence the gut microbiota. *Wolbachia* may lead to decreased microbial diversity due to competitive behavior [54], which may contribute to the lower diversity of gut microbiota in PFM than that of OFM. In *Drosophila melanogaster*, *Wolbachia* can reduce the richness of *Acetobacter* [55], but this group was not at a low abundance in PFM. Whether *Wolbachia* in PFM influences other microbiota requires a comparison of *Wolbachia* infected and *Wolbachia* free lines, which might be generated through antibiotic treatment or by taking advantage of natural polymorphism in infection status within natural populations [56].

### 4.4. Implications for Pest Management

The insect-associated microbes provide new targets for developing novel pest control methods [6,18,57,58]. The first step to find potential bacterial targets is to investigate the bacterial community, its impact on the pests, and its stability. We found that the community of the gut microbiota similar within moth species in spite of host fruit differences for microbes such as *Pseudomonas*, *Pantoea*, *Lactobacillus*, *Gluconobacter*, and *Acetobacter*. Among the abundant bacteria taxa, *Pseudomonas brenneri* plays a prominent role in the removal of heavy metals [59]. This species is significantly more abundant in OFM than PFM. *Gluconobacter cerinus* was another species present in PFM and OFM, which may have a beneficial role as in the case of fruit flies where it can affect reproduction [60]. *Pantoea* is a highly diverse genus that can cause plant diseases and human diseases but also have functions in habitat restoration and pesticide degradation [61]. Functional studies of these bacteria may help to identify potential targets for developing control methods of these two fruit moths.

The similar composition of the gut microbes may be related to the horizontal spread of microbes through host plants [20,62], or vertical transmission generation by generation, which need further investigation. The similar composition may also indicate functions related to the common biology of both species, particularly in terms of the fruit-feeding larvae. These larvae bore into fruit or shoots soon after egg hatching, reducing their likelihood of exposure to environmental bacteria when compared to the leaf-feeding moths. In the fruit-feeding spotted wing drosophila, Drosophila suzukii, the gut microbiota provides nutrition by providing protein for their hosts [63]. Larvae of fruit moths often feed on immature fruits, which are rich in compounds such as organic acids and tannins. The tannins are endogenous inhibitors of the growth of numerous species of pests by negatively effecting the metabolism of insects [64]. Sap sucking pests such as whiteflies and aphids make use of symbionts for their essential amino acids [65,66]. Symbiotic essential amino acids provisioning or supplementation has also been found in the American cockroach and Cerambycid [67,68]. Whether the gut bacterial microbes of PFM and OFM are involved in nutrition provisioning or supplementation needs future investigation. There are examples of gut microbiota in lepidopteran hosts helping to detoxify host toxins [69,70], but whether the fruit moths need microbes to help them to detoxify defensive chemicals is unclear. Further tests of such hypotheses may provide insights into the development of novel control approaches.

## 5. Conclusions

In the study, we compared gut bacterial microbiota of two co-occurring agricultural pests, the PFM and the OFM, collected from different orchards and host plant species. We found the two fruit moths share many features of gut microbiota even when they use different host plants. Environment of orchards can influence the gut microbiota of the two fruit moths.

## Figures and Tables

**Table 1:** Samples of the peach fruit moth (PFM), *Carposina sasakii*, and the oriental fruit moth (OFM), *Grapholita molesta*, used in the study.

Code *	Species	Collecting Location	Host (code)	Coordinate	NO.
PKPR	PFM	Kaosanji of Pinggu district (K)	Pear (PR)	40°12′ N, 117°19′ E	5
OKPR	OFM				5
PLPR	PFM	Lvfulong of Yanqing district (L)	Pear (PR)	40°32′ N, 116°4′ E	5
OLPR	OFM				7
PGAE	PFM	Liugou of Yanqing district (G)	Apple (AE)	40°27′ N, 116°6′ E	4
OGAE	OFM				6
PDAE	PFM	Dafengying of Yanqing district (D)	Apple (AE)	40°26′ N, 115°54′ E	5
OYPH	OFM	Linguosuo of Haidian district (Y)	Peach shoot (PH)	39°58′ N, 116°13′ E	5
OSPH	OFM	Shuangxin of Haidian district (S)	Peach shoot (PH)	39°57′ N, 116°12′ E	2

NO., the number of individuals used for 16S rRNA gene sequencing. *, the first letter in the code represents the species (P, PFM; O, OFM), the second letter represents the collection site, while the third and fourth letters represent the host (PR, pear; AE, apple; PH, peach shoot).

## Supplementary Materials

The following are available online at https://www.mdpi.com/2075-4450/11/12/840/s1. Table S1: Basic statistics on 16S rRNA gene sequencing of the gut bacterial microbiota for the peach fruit moth (PFM) *Carposina sasakii* and the oriental fruit moth (OFM) *Grapholita molesta* using Illumina Miseq platform. * primers used for sequencings. Table S2: Statistics on number of OTUs and their classification identified after filtering for the peach fruit moth (PFM) *Carposina sasakii* and the oriental fruit moth (OFM) *Grapholita molesta*. Table S3: Alpha diversity of the gut bacterial microbiota for the peach fruit moth (PFM) *Carposina sasakii* and the oriental fruit moth (OFM) *Grapholita molesta*. Figure S1: Microbial composition identified in the peach fruit moth (PFM) *Carposina sasakii* and the oriental fruit moth (OFM) *Grapholita molesta* when *Wolbachia* were kept in analysis. Figure S2: Rarefaction curves depicted from original sequencing data sets and randomly subsampled data sets with the same number of 16S rRNA sequences determined from the peach fruit moth (PFM), *Carposina sasakii*, and the oriental fruit moth (OFM) *Grapholita molesta*.
