# Supplementary material for: Similar Gut Bacterial Microbiota in Two Fruit-Feeding Moth Pests Collected from Different Host Species and Locations
Source: Insects. 2020 Nov 28;11(12):840. doi: 10.3390/insects11120840 (PMC7759971; doi:10.3390/insects11120840)
Supplement: Supplementary file 1 [file insects-11-00840-s001.pdf]

Table S1 Basic statistics on 16S rRNA gene sequencing of the gut bacterial microbiota for the peach fruit moth (PFM) *Carposina sasakii* and the oriental fruit moth (OFM) *Grapholita molesta* using Illumina Miseq platform. * primers used for sequencings.

Item	Data
	338F-806R*
	5′-
	ACTCCTACGGGAGGCAGCAG-
	3′
Amplified region	5′-
Primer sequence 338F	GGACTACHVGGGTWTCTAAT-
Primer sequence 806R	3′
Samples number	44
Passed QC (bp)	905462037
Average length (bp)	418.2527697
Total raw reads	2164868
Average reads	49201
Minimum read count	33359
Maximum read count	71332
Total chloroplast read	206585
Total mitochondrial read	14499
Total *Wolbachia* read	549604
Average reads (chloroplast & mitochondrial & *Wolbachia* sequence removed)	17516
Minimum read count (chloroplast & mitochondrial & *Wolbachia* sequence removed)	712
Maximum read count (chloroplast & mitochondrial & *Wolbachia* sequence removed)	66707
Effective sequence count per sample (after filtering)	385
Delete the total number of reads	2147928

Table S2 Statistics on number of OTUs and their classification identified after filtering for the peach fruit moth (PFM) *Carposina sasakii* and the oriental fruit moth (OFM) *Grapholita molesta*.

Group	Sample	Phylum	Class	Order	Family	Genus	Species	OTU
ALL	44	12	21	43	83	158	208	239
PFM	19	5	11	25	40	61	78	87
OFM	25	9	19	42	77	131	174	199

Table S3 Alpha diversity of the gut bacterial microbiota for the peach fruit moth (PFM) *Carposina sasakii* and the oriental fruit moth (OFM) *Grapholita molesta*. Samples were numbered based on their population codes. See Table 1 for population codes.

Species	Sample	Sobs	Shannon	Simpson	Ace	Chao	Coverage
PFM	CDAE04	14	0.8042	0.5999	27.3333	23.3333	0.9792
	CDAE09	72	3.5761	0.0422	101.7201	91.5000	0.9299
	CDAE12	10	1.0715	0.4302	21.6389	15.0000	0.9870
	CDAE13	12	1.2048	0.3780	128.3436	22.5000	0.9818
	CDAE24	14	1.2353	0.4190	20.9830	17.3333	0.9870
	CGAE01	15	0.9945	0.4643	99.6677	37.5000	0.9740
	CGAE04	16	0.9044	0.4968	58.0000	38.0000	0.9688
	CGAE08	37	1.2538	0.6123	55.4515	54.0000	0.9558
	CGAE09	70	3.3459	0.0744	102.1997	99.0769	0.9273
	CKPR04	17	1.8504	0.1994	42.5192	20.7500	0.9844
	CKPR09	23	2.2067	0.1538	52.0397	41.0000	0.9766
	CKPR11	23	1.3267	0.4207	35.3932	32.0000	0.9740
	CKPR12	13	1.0590	0.5491	16.5739	16.0000	0.9896
	CKPR20	12	1.4081	0.3183	29.2656	22.0000	0.9870
	CLPR03	39	2.0336	0.2745	117.3853	60.1111	0.9481
	CLPR05	65	3.2354	0.0829	74.7984	72.0588	0.9584
	CLPR12	76	3.5022	0.0541	112.8846	117.3333	0.9169
	CLPR13	88	3.7448	0.0400	136.1734	134.8667	0.9013
	CLPR14	68	2.8322	0.1399	190.0777	123.5000	0.9039
OFM	GGAE01	20	2.0203	0.1648	64.9119	29.0000	0.9766
	GGAE02	15	1.0927	0.4477	63.2108	33.0000	0.9766
	GGAE03	36	2.1105	0.2211	78.5352	58.6667	0.9558
	GGAE06	14	0.9943	0.4742	53.0358	28.0000	0.9792

GGAE09	11	1.2358	0.3804	21.4545	21.0000	0.9870
GGAE11	12	1.0601	0.4485	29.7927	22.5000	0.9818
GKPR11	19	1.7760	0.2400	50.5710	23.2000	0.9818
GKPR12	27	2.2625	0.1650	36.1313	31.6667	0.9792
GKPR13	25	2.2548	0.1408	34.9125	34.0000	0.9766
GKPR19	16	1.5763	0.3494	21.2953	26.0000	0.9870
GKPR24	7	0.4886	0.7465	17.0000	10.0000	0.9896
GLPR01	26	1.7019	0.2918	52.0495	39.2000	0.9688
GLPR09	32	2.1643	0.1870	52.8320	45.0000	0.9636
GLPR12	39	1.9638	0.3240	131.9933	91.5000	0.9455
GLPR13	26	1.5542	0.3259	66.8079	56.3333	0.9636
GLPR14	57	2.6901	0.1722	75.7204	70.5714	0.9481
GLPR15	17	1.1179	0.4572	28.9241	24.0000	0.9792
GLPR17	32	1.5102	0.3753	126.4096	84.5000	0.9455
GSPH06	10	0.5320	0.7691	18.5000	15.0000	0.9870
GSPH09	8	0.8914	0.5201	12.5625	9.5000	0.9922
GYPH07	6	0.8359	0.5150	6.0000	6.0000	1.0000
GYPH12	6	0.7292	0.5539	27.4063	9.0000	0.9922
GYPH14	16	1.1227	0.4731	50.1985	30.0000	0.9792
GYPH19	8	0.7389	0.5754	10.4563	9.5000	0.9922
GYPH28	12	1.8464	0.2214	13.7867	12.5000	0.9948

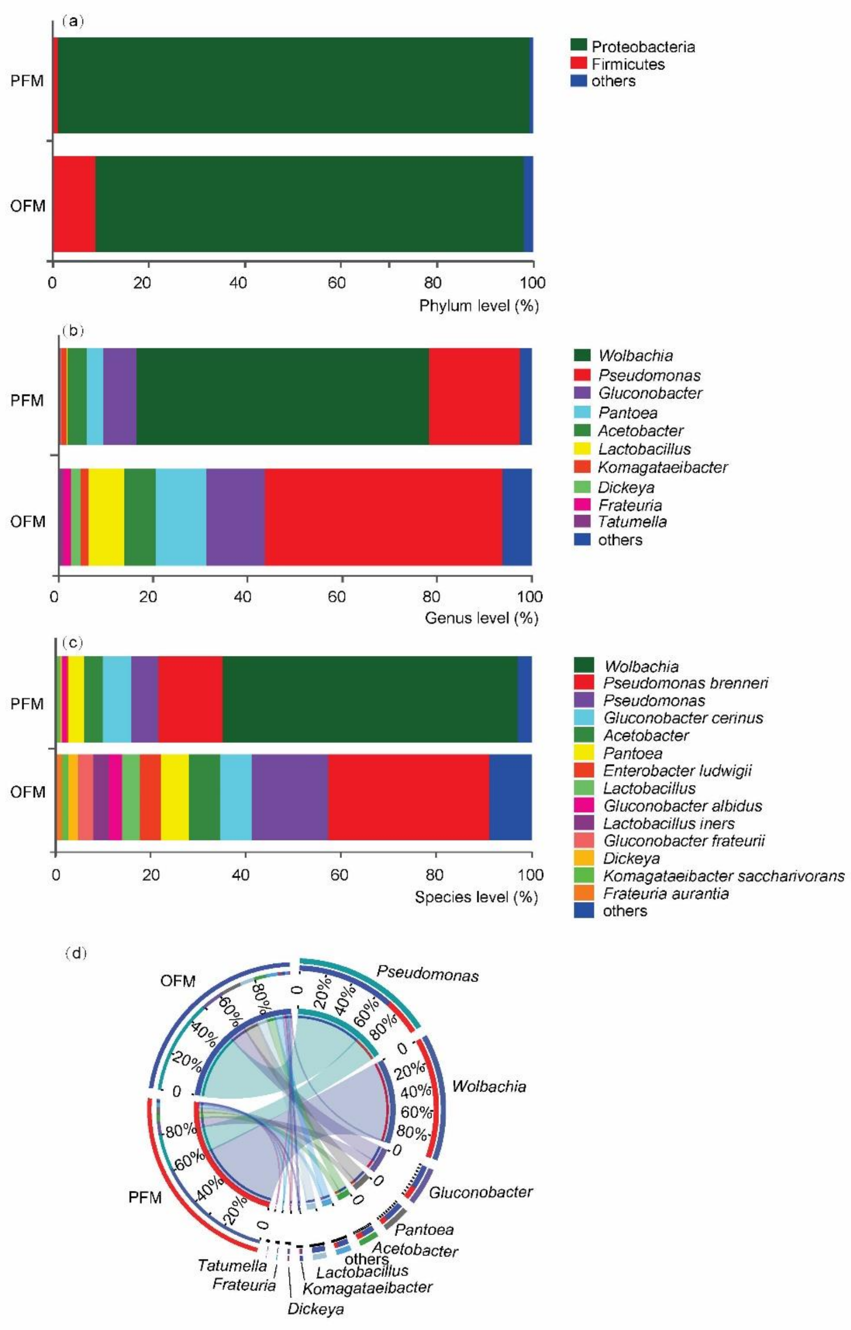

Figure S1 Microbial composition identified in the peach fruit moth (PFM) *Carposina sasakii* and the oriental fruit moth (OFM) *Grapholita molesta* when *Wolbachia* were kept in analysis. Community composition of the microbiome on phylum (a), genus (b), and species (c) levels for the OFM and PFM. (d) The cooccurrence relation graph describes the abundance of correspondence between samples and species. Each unit was represented by one color. At the genus level, OTUs of PFM were mainly annotated to *Wolbachia* (62.06%), *Pseudomonas* (19.09%), *Gluconobacter* (6.98%), *Acetobacter* (4.05%), and *Pantoea* (3.59%), while OTUs of OFM were mainly annotated to *Pseudomonas* (49.96%), *Gluconobacter* (12.53%), *Pantoea* (10.70%), *Lactobacillus* (7.65%), *Acetobacter* (6.61%).

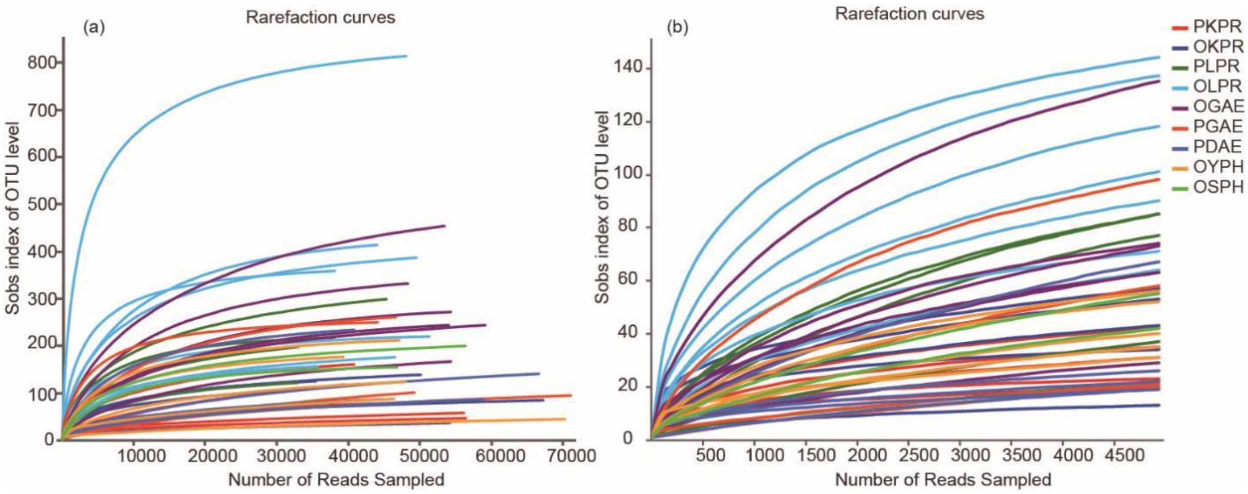

Figure S2 Rarefaction curves depicted from original sequencing data sets and randomly subsampled data sets with the same number of 16S rRNA sequences determined from the peach fruit moth (PFM), *Carposina sasakii*, and the oriental fruit moth (OFM) *Grapholita molesta*. (a) Rarefaction curves of original sequencing samples. (b) Rarefaction curves of subsampled sequences. The subsampled data were sampled based on the sample with the lowest sequencing depth. OTUs with a total sequence number greater than or equal to 20 and OTUs present in at least 3 samples with a sequence number greater than or equal to 5 were used for subsampling. Both the raw data and the subsampled data start to plateau at about 5000 reads. See Table 1 for population code.
